# Small Molecule Proprotein Convertase Inhibitors for Inhibition of Embryo Implantation

**DOI:** 10.1371/journal.pone.0081380

**Published:** 2013-12-04

**Authors:** Huiting Ho, Harmeet Singh, Sophea Heng, Tracy L. Nero, Sarah Paule, Michael W. Parker, Alan T. Johnson, Guan-Sheng Jiao, Guiying Nie

**Affiliations:** 1 Prince Henry's Institute of Medical Research, Clayton, Victoria, Australia; 2 Department of Biochemistry and Molecular Biology, Monash University, Clayton, Victoria, Australia; 3 Biota Structural Biology Laboratory and ACRF Rational Drug Discovery Centre, St Vincent's Institute of Medical Research, Fitzroy, Victoria, Australia; 4 Department of Biochemistry and Molecular Biology, Bio21 Molecular Science and Biotechnology Institute, The University of Melbourne, Parkville, Victoria, Australia; 5 Department of Chemistry, PanThera Biopharma LLC, Aiea, Hawaii, United States of America; State Key Laboratory of Reproductive Biology, Institute of Zoology, Chinese Academy of Sciences, China

## Abstract

Uterine proprotein convertase (PC) 6 plays a critical role in embryo implantation and is pivotal for pregnancy establishment. Inhibition of PC6 may provide a novel approach for the development of non-hormonal and female-controlled contraceptives. We investigated a class of five synthetic non-peptidic small molecule compounds that were previously reported as potent inhibitors of furin, another PC member. We examined (i) the potency of these compounds in inhibiting PC6 activity *in vitro*; (ii) their binding modes in the PC6 active site *in silico*; (iii) their efficacy in inhibiting PC6-dependent cellular processes essential for embryo implantation using human cell-based models. All five compounds showed potent inhibition of PC6 activity *in vitro*, and *in silico* docking demonstrated that these inhibitors could adopt a similar binding mode in the PC6 active site. However, when these compounds were tested for their inhibition of decidualization of primary human endometrial stromal cells, a PC6-dependent cellular process critical for embryo implantation, only one (compound 1o) showed potent inhibition. The lack of activity in the cell-based assay may reflect the inability of the compounds to penetrate the cell membrane. Because compound's lipophilicity is linked to cell penetration, a measurement of lipophilicity (logP) was calculated for each compound. Compound 1o is unique as it appears the most lipophilic among the five compounds. Compound 1o also inhibited another crucial PC6-dependent process, the attachment of human trophoblast spheroids to endometrial epithelial cells (a model for human embryo attachment). We thus identified compound 1o as a potent small molecule PC6 inhibitor with pharmaceutical potential to inhibit embryo implantation. Our findings also highlight that human cell-based functional models are vital to complement the biochemical and *in silico* analyses in the selection of promising drug candidates. Further investigations for compound 1o are warranted in animal models to test its utility as an implantation-inhibiting contraceptive drug.

## Introduction

The proprotein convertases (PCs) are a family of nine serine proteases implicated in the processing of a multitude of precursor proteins [1], [2]. The first seven members [PC1/3, PC2, furin, PACE4, PC4, PC5/6 (to be referred as PC6 in this report) and PC7] activate a large number of polypeptide hormones, growth factors, adhesion molecules, various viral surface proteins and pro-toxins of bacteria by cleavage at basic residues [2]. The eighth and ninth members (SKI-1 and PCSK9) do not require a basic residue for cleavage and they play major roles in regulation of lipid homeostasis [2], [3]. Accumulated evidence over the last decade has confirmed PCs as potential therapeutic targets for several important pathologies including osteoarthritis, cancer, cardiovascular disease and viral infections [1]. Therefore, development of PC inhibitors is clearly an important research and development field.

Our interest in PC inhibitors originated from studies aiming at inhibiting PC6 in the female reproductive tract to inhibit embryo implantation. Uterine PC6 is pivotal in embryo implantation and is essential for the establishment of pregnancy [4]. To enable implantation, the uterus must acquire epithelial receptivity and undergo a process known as decidualization to differentiate stromal fibroblasts into phenotypically and functionally distinct decidual cells [5]. We have previously shown that PC6 is critical for both uterine epithelial receptivity and stromal cell decidualization [6], [7], [8], [9]. Knockdown of PC6 in a human endometrial epithelial cell line HEC1A significantly reduced its receptivity for blastocyst adhesion [6]. Decidualization of primary human endometrial stromal cells (HESCs) was inhibited when PC6 activity was blocked [8], [10]. It has also been demonstrated in mice that when uterine PC6 production was blocked, decidualization was inhibited and implantation was prevented [11]. In addition, PCs including PC6 also play an important role in HIV infection [12], [13], [14]. Therefore, inhibition of PC6 is an attractive approach to develop novel, non-hormonal and female-controlled contraceptives that could also protect women from HIV infection.

The majority of PC inhibitors reported in the literature to date have been proteins or peptides [15]. Nona-D-arginine (Poly R) is one of the most potent peptide based PC inhibitors known to date. Poly R inhibits PC6 *in vitro* with a Ki in the nanomolar range and has been shown to inhibit HIV in cell culture [16], [17]. We have previously demonstrated that Poly R inhibits decidualization of HESC in culture and have evaluated the therapeutic potential of a PEGylated Poly R [covalently attached with polyethylene glycol (PEG) polymers] in inhibition of implantation in rabbits [8], [10]. However, the physiochemical properties of Poly R could limit their usefulness in therapeutic applications in women. Therefore, we continue to search for potent PC6 inhibitors with the desired characteristics such as serum stability and cell permeability.

In this study, we evaluated five synthetic small molecule compounds derived from 2,5-dideoxystreptamine chemical scaffold previously reported by Jiao *et al.*, 2006 [15]. Four of these compounds (1e, 1f, 1g, 1n) were previously shown to be potent inhibitors of both human furin and PC6 *in vitro* [15]. Compound 1o was shown to be a relatively poor inhibitor of furin but no data on PC6 was reported [15]. Here, the inhibitory potency of all five compounds against human PC6 (hPC6) was determined *in vitro*. *In silico* docking studies were performed to visualise the potential binding mode of these inhibitors in the active site of hPC6 and to gain an understanding of how this may relate to their inhibitory activity. The therapeutic potential of these small molecule inhibitors was then examined in *in vitro* human cell-based models to investigate their ability to inhibit two important PC6-mediated cellular processes essential for embryo implantation: (1) decidualization of primary HESCs and (2) attachment of human trophoblast spheroids (surrogate for embryos) to endometrial epithelial cells.

## Materials and Methods

### Small molecule PC inhibitors

**Figure 1:**
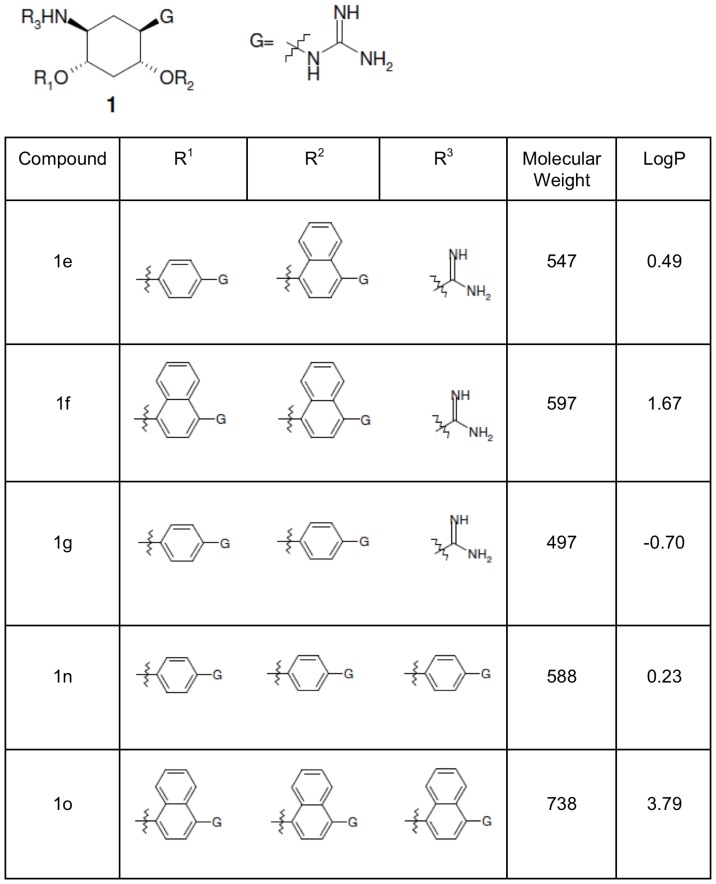
The chemical structure, molecular weight and logP values for the 2,5-dideoxystreptamine-derived small molecules. LogP values were calculated using ACDlabs Structure Designer Suite logP software.

Small molecule compounds 1e, 1f, 1g, 1n and 1o (Figure 1), derived from 2,5-dideoxystreptamine, were synthesized as previously reported by Jiao, *et al*. [15].

### Cell culture

Ishikawa (Clone 3-H-12), a human endometrial adenocarcinoma cell line of epithelial origin, was kindly provided by Dr. Masato Nishida (National Kasumigaura Hospital, Ibarak, Japan) [18]. JAR cells, a human choriocarcinoma cell line, was purchased from ATCC (American Type Culture Collection). Ishikawa and JAR cells were cultured in phenol-red MEM without glutamine and RPMI 1640 Medium, GlutaMAX™, respectively, supplemented with 10% charcoal stripped fetal bovine serum (CS-FBS, Thermo Electron Corporation, Maple Plain, MN) and 100 µg/ml streptomycin and 100 IU/ml penicillin (Gibco®, Mulgrave, VIC, Australia). Ishikawa cells were used within 10 passages according to the provider's recommendation to avoid changes in cell characteristics such as down-regulation of estrogen receptor and progesterone receptor expression.

### Inhibition of PC6 activity by small molecule compounds

An *in vitro* PC6 activity assay as described previously [8], [9] was used to evaluate PC6 inhibition by small molecule compounds. In brief, 2 units of active recombinant human PC6 (rhPC6) (PhenoSwitch BioScience Inc., Quebec, Canada) were incubated with small molecule compounds (10 µM), in Dulbecco's modified Eagle's medium/Ham's F12 culture medium (DMEM/F12, Sigma, St. Louis, MO) in the presence of 100 µM fluorogenic substrate pERTKR-AMC (Bachem, Torrance, CA) at 37°C. The real-time kinetic progression of substrate hydrolysis [release of fluorescent 7-amino-4-methylcoumarin (AMC)] was monitored every 5 min at excitation/emission of 355/460 nm (Wallac, Victor 2 spectrophotometer; PerkinElmer, Boston, MA) for 1 h. Inhibition of PC6 activity was expressed as a reduction in the rate of substrate hydrolysis relative to the control (PC6 activity in the absence of inhibitory peptides). At least two independent experiments were performed for each compound.

### In silico docking of small molecule compounds into the catalytic site of hPC6

As no crystal structure of PC6 from any species is available, a homology model of hPC6 was used for the *in silico* docking studies. The construction of the hPC6 homology model has been previously reported [19]. The hPC6 active site is a canyon-like groove lined with clusters of negatively charged groups that are classified into sub-pockets that are defined as S1-S6 and S1′ [20]. The important residues in each sub pockets are S1 – D277, D325; S2 – D173, E210; S3 – L246, W273; S4 – E255, D283; S5 – D276, D283; S6 – D249, D252; S1′ – K212, R216, H381 [20]. The hPC6 catalytic triad consists of D172, H213 and S385 [20]. The five compounds were constructed using standard bond lengths and bond angles within SYBYL-X 2.0 (Certara L.P., http://www.tripos.com) and then structurally optimized using the MMFF94s forcefield and partial atomic charges, conjugate gradient convergence method; termination of the optimization was achieved when the gradient difference of successive steps was <0.05 kcals/mol Å (all other parameters were at default values). Docking of the compounds into the catalytic domain of the hPC6 homology model was carried out using Surflex v2.6. The protomol was generated using the automated method, a threshold of 0.50 and a bloat value of 2. The GeomX mode was used, all other parameters were at default values. The C-Score function was used to rank the docked compound poses, the top twenty ranked poses for each compound were examined visually. Docked poses of compounds 1g and 1o have been chosen to illustrate how these compounds can bind into the hPC6 active site.

### Decidualization of HESCs and inhibition by small molecule compounds

Human endometrial tissues were obtained from non-pregnant women undergoing curettage following laparoscopic sterilization or assessment of tubal patency. Ethical approval was granted by the Human Ethics Committee of Southern Health, Melbourne, Australia and written informed consent was obtained from all tissue donor patients. Tissues collected between Day 8–24 were processed within 24 h. Human endometrial stromal cells (HESCs) were isolated by enzymatic digestion and filtration as previously described [8], [19], [21]. HESCs (>97%) were cultured in T25 cm^2^ flasks in DMEM/F12 medium supplemented with 10% CS-FBS, 2 mM L-glutamine (Sigma), 100 µg/ml streptomycin and 100 IU/ml penicillin [22]. Once 70–80% confluent, the HESCs were passaged into 12-well plates (8×10^8^ cells/well) and cultured to 80% confluence. For decidualization, cells were treated with estradiol 17-β (E2, 10^−8^ M), medroxy-progesterone acetate (MPA, 10^−7^ M) and 8-bromoadenosine 3′:5′ cyclic monophosphate (camp, 5×10^−4^ M) (all from Sigma) for 72 h in serum free DMEM/F12 containing 0.1% BSA. Decidualization success was confirmed by a significant increase in the decidual markers prolactin (PRL) in the conditioned medium by ELISA (Bioclone Australia Pty Ltd., Sydney, Australia) as per the manufacturer's instructions [8]. To access decidualization inhibition by the small molecule compounds, HESCs were decidualized in the absence (control) or presence of 10 µM of each compound for 72 h with the media replaced every 24 h. Compound 1o was also tested for dose-dependent inhibition at 1 and 5 µM. The time course of inhibition of decidualization was expressed as a percentage reduction in prolactin levels in the conditioned media relative to the control. The levels of an additional decidual marker insulin-like growth factor binding protein-1 (IGPBP-1) in the media were also measured by ELISA (RayBiotech, Norcross, GA) according to the manufacturer's instructions. Three independent experiments were performed using different cell preparations for each experiment. P<0.05 was considered statistically significant.

### Lipophilicity calculation

The logP value is a measure of the lipophilicity of a compound; the larger the logP value, the more lipophilic the compound is. Lipophilicity (or hydrophobicity) is linked to the compound's ability to penetrate the cell membrane; if a compound is too hydrophilic then it will not be able to cross the cell membrane and if it is too lipophilic, it may remain in the membrane and not pass through into the cell. The logP of each compound in Figure 1 was calculated using ACDlabs Structure Designer Suite logP software (http://www.acdlabs.com).

### In vitro human trophoblast spheroid attachment assay

The in vitro efficacy of compound 1o to inhibit embryo attachment was determined using a human trophoblast spheroid attachment model involving the co-culture of trophoblast JAR spheroids and monolayers of Ishikawa endometrial epithelial cells [23].

To generate JAR spheroids, JAR cells were grown in suspension in culture media (10 ml) at a density of 2.5×10^5^ cells/ml in T75 Nunc tissue culture flask with rocking at a speed of 50 rpm (ERPM4, Ratek Instruments, Victoria, Australia) for 20 – 22 h. Selection of JAR spheroids of size similar to human blastocyst were done as described previously [23]. The spheroid suspension was passed first through a cell strainer (BD Bioscience, NSW, Australia) with sieve size 100 µm, to eliminate large cell aggregates, then through a cell strainer of 70 µm sieve size to capture spheroids of size between 70 and 100 µm. Ishikawa cells (1.5×10^4^ cells/well) were cultured in 96-well plates with or without compound 1o (5 µM or 10 µM) for 3 days to form a cell monolayer, media was then replaced with 50–100 spheroids/well in 100 µl media, and the Ishikawa monolayer and spheroids were co-cultured for 1 h in an atmosphere of 5% CO_2_ at 37°C. Loosely attached spheroids were removed by washing twice with phosphate-buffered saline (PBS), first with 200 µl and second with 100 µl. The percentage of attachment (attached/seeded spheroids) was calculated and the data was presented in relative to control. Data presented are from four duplicate wells and three independent experiments.

### Western blot analysis of Pro- integrin-αV cleavage

Integrin-αV in the human endometrial epithelium is initially synthesized as a non-functional proform, PC6 is recently shown to cleave pro-integrin-αV into its functional heavy and light chains during the establishment of endometrial receptivity [24]. To determine whether compound 1o inhibits the PC6 cleavage of pro-integrin-αV, total protein lysates were extracted from Ishikawa cells treated with vehicle or different doses of compound 1o, by lysing the cells with a lysis buffer [50 mM Tris/HCl, pH 7.4; 150 mM NaCl; 1% (vol/vol) Triton X-100; 1 mM EGTA and 2 mM EDTA] containing protease inhibitor cocktail (Pierce, Rochford, IL, USA). Total protein (20 µg) were resolved on 7.5% SDS-polyarcylamine gels. Integrin αV antibodies were selected according to their recognition sites; Q-20 (1∶250, Santa Cruz Biotechnology, Santa Cruz, CA, USA) for the detection of the heavy chain and Ab1930 (1∶300; Millipore) for the proform. Secondary rabbit-HRP antibody (Dako, Victoria, Australia) was used. Membranes were probed with GAPDH-HRP monoclonal antibody (1∶1000; Cell Signaling) for loading control. Bands were visualised using a Lumi-light system (Roche, Germany). Densitometric analysis of band intensity was performed using ImageJ.

### Comparison of compound 1o and a potent peptide PC6 inhibitor in the inhibition of decidualisation and endometrial epithelial cell receptivity

The inhibition of decidualisation of primary HESCs by compound 1o was compared to that of a known peptide PC6 inhibitor, C-30k-PEG Poly R (synthesised by Mimotopes, Clayton, Australia), which was previously proved to potently inhibit decidualisation of primary HESCs [9]. The effect of C-30k-PEG Poly R on Ishikawa cell receptivity to trophoblast spheroids was also examined using the *in vitro* human trophoblast spheroid attachment assay as described above, and the results were compared to that of compound 1o.

## Results

### The five small molecule compounds are potent PC6 inhibitors in vitro

**Figure 2:**
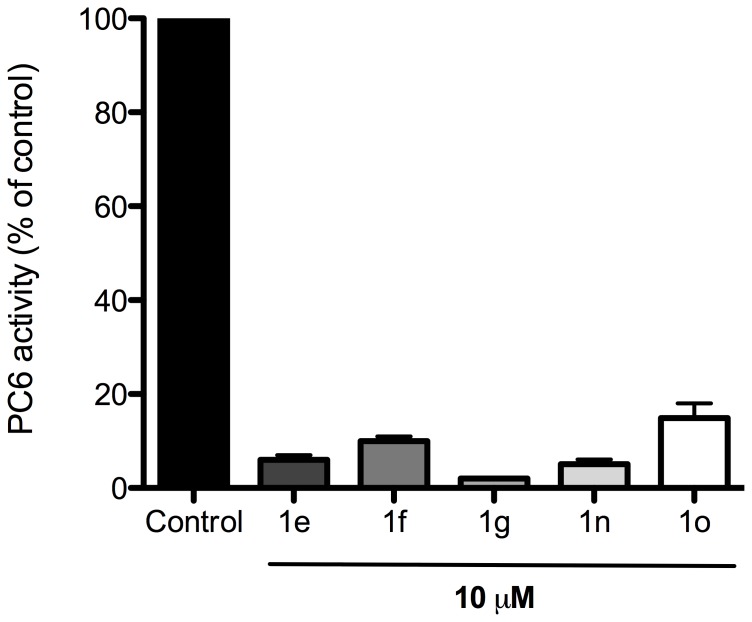
Inhibition of PC6 activity by small molecule compounds. The data are expressed as percent reduction of PC6 activity based on the rate of substrate hydrolysis relative to the control. Each value represents mean ± SEM of at least two independent experiments.

Of the five compounds, four of them (1e, 1f, 1g and 1n) were previously shown to be potent inhibitors of both human furin and PC6 *in vitro* [15] but compound 1o had not been screened against PC6. We confirmed that all five compounds inhibit *in vitro* rhPC6 hydrolysis of the fluorogenic peptide substrate pERTKR-AMC [19]. At 10 µM, compounds 1e, 1f, 1g and 1n inhibited rhPC6 ≥ 90%, whereas compound 1o had a slightly lower inhibitory potency of 85% (Figure 2).

### In silico docking reveals a similar binding mode for all five compounds in the hPC6 active site

**Figure 3:**
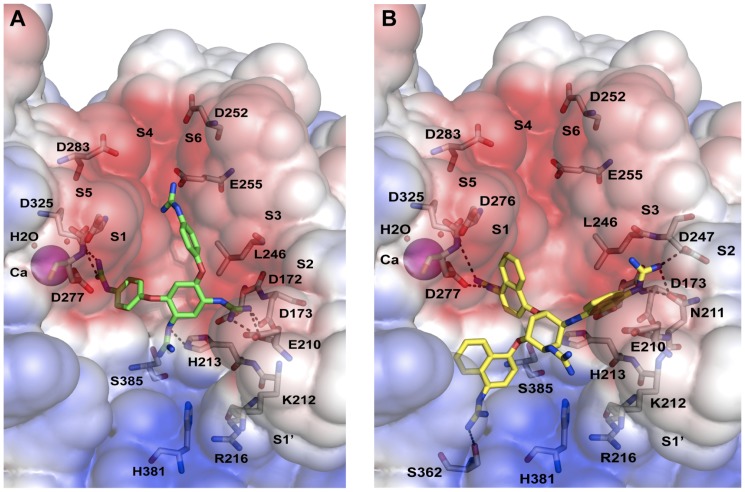
Putative binding modes of compounds 1g and 1o in the active site of hPC6. hPC6 is depicted as a molecular surface colored by electrostatic potential (red – regions of negative potential, blue – regions of positive potential), selected residues of the S1–S6, S1′ sub-pockets and catalytic triad are shown as sticks. The Ca^2+^ cation close to the active site is shown as a magenta sphere (labelled as Ca) and the coordinated waters as small red spheres (labelled as H2O). The location of sub-pockets S1–S6 and S1′ are indicated. Electrostatic/hydrogen bonds/polar contacts are indicated by black dashed lines. (A) Binding mode of compound 1g (green colored sticks). The four guanidino moieties are located in S1, S2 and S4 sub-pockets and near the catalytic triad (D172, H213, S385). (B) Binding mode of compound 1o (yellow colored sticks). The four guanidino moieties are located in the S1, S2 and S3 sub-pockets and near the catalytic triad (D172, H213, S385).

Putative binding modes of compounds 1g and 1o are shown in Figure 3. The hPC6 active site is a long groove able to accommodate hexa-peptide length compounds. The small molecule inhibitors, compounds (1e, 1f, 1g, 1n and 1o), need to be able to “hook” themselves into the hPC6 active site via strong interactions with the negatively charged residues that line this site. The five compounds contain these “hooks” in the form of four guanidino moieties. The four guanidino substituents on the 2,5-dideoxystreptamine ring can adopt a variety of conformations within the hPC6 active site; however the 2,5-dideoxystreptamine ring is physically restricted to the triangular region connecting sub-pockets S1, S2 and S1′ (Figure 3).

The binding modes depicted in Figure 3 for compounds 1g and 1o block access to the catalytic site of hPC6. The electrostatically positive guanidino moieties of the compounds are able to interact with the negatively charged residues lining the sub-pockets of the hPC6 active site (these are the red regions in Figure 3). The compounds can also make numerous hydrogen bonds, polar contacts and π-π stacking interactions with hPC6 active site residues. The G, R_1_, R_2_ and R_3_ substituents of the di-aryl 2,5-dideoxystreptamine compounds 1e, 1f and 1g (Figure 1) can occupy one or more of the sub-pockets S1, S2 and S4 and also the region near the catalytic triad (D172, H213, S385, Figure 3A). In contrast, the G, R_1_, R_2_ and R_3_ substituents of the tri-aryl 2,5-dideoxystreptamine compounds compounds 1n and 1o (Figure 1) are able to occupy the S1, S2 and S3 sub-pockets, in addition to the region near the catalytic triad (Figure 3B). Compound 1n can also occupy the S4 sub-pocket; however, the physical size of the 3 naphthyl rings prevent compound 1o from doing so.

The binding modes for the five compounds (1e, 1f, 1g, 1n and 1o) in the hPC6 active site were consistent with the binding mode of compound 1n in human furin described previously by Jiao *et al* [15].

### Only compound 1o inhibits decidualization of HESCs

**Figure 4:**
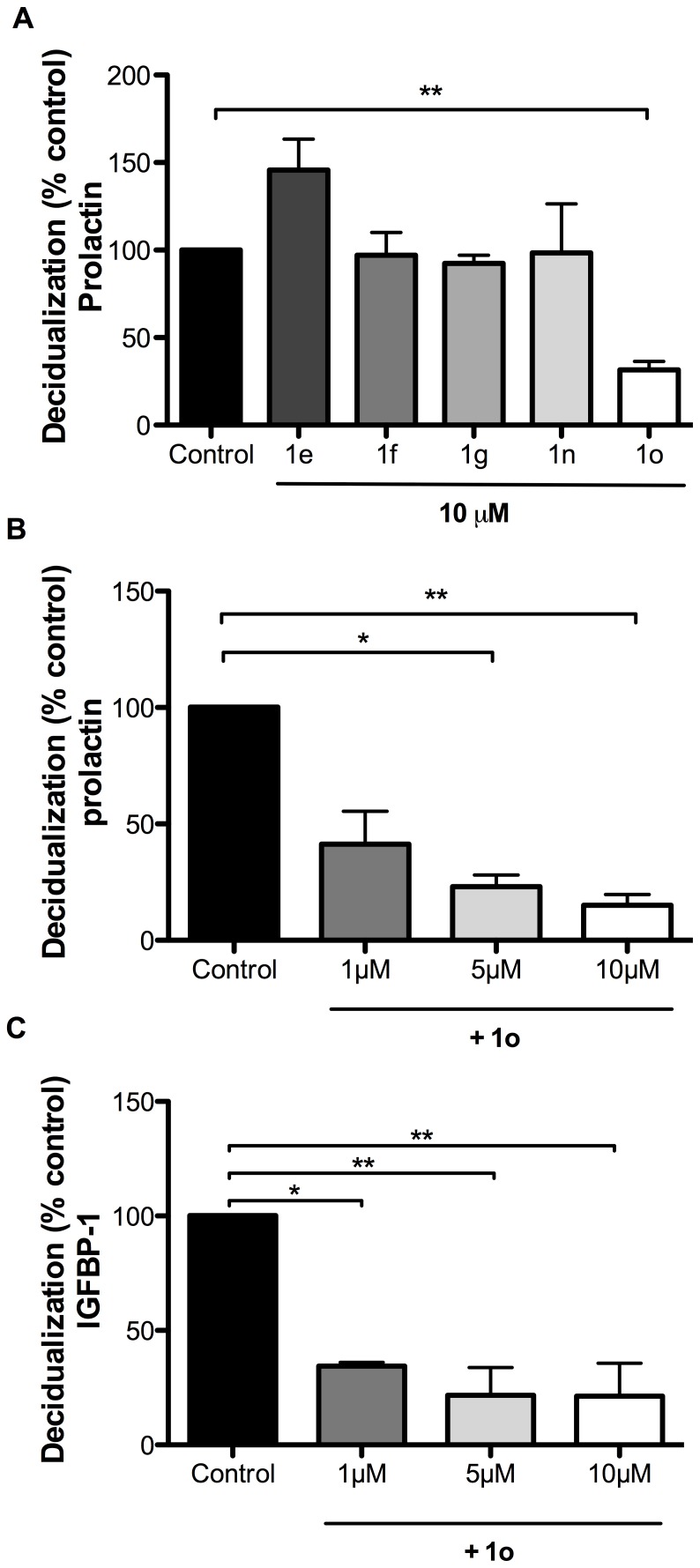
Inhibition of decidualization of HESCs. (A) Inhibition of decidualization by the five compounds at 10 µM using prolactin as the decidual marker. (B) Dose-dependent inhibition of decidualization by compound 1o using prolactin as the decidual marker. (C) Confirmation of decidualization inhibition by compound 1o with an additional decidual marker, IGFBP-1. The data are expressed as percentage reductions relative to control (no inhibitors). Each value represents mean ± SEM of three independent experiments. *P<0.05; **P<0.01

Decidualization of HESCs is a cellular process essential for embryo implantation. PC6 is critical for decidualization and blocking of PC6 activity inhibits the process [8], [25]. To determine whether the five compounds would also inhibit PC6-dependent decidualization, HESCs were cultured without (control) or with 10 µM of each compound in the presence of decidualizing stimuli [8]. Using prolactin as the decidual marker, of the five compounds, only compound 1o significantly inhibited decidualization, whereas the other four compounds had no effect (Figure 4A). Further experiments showed that compound 1o inhibited decidualization in a dose-dependent manner, inhibiting ∼60% at 1 µM, ∼80% at 5 µM and ∼85% at 10 µM (Figure 4B). Inhibition of decidualisation by compound 1o was also confirmed by a significant decrease in the level of an additional decidual marker, IGFBP-1 (Figure 4C).

### Compound 1o is the most lipophilic of the five compounds

Lipophilicity is a major determining factor in a compound's pharmaceutical properties such as penetration across cellular membranes. We therefore calculated the lipophilicity (logP) of the five compounds (Figure 1). Based on their predicted logP values, the relative order of lipophilicity is: compound 1o (moderately lipophilic) > compound 1f > compound 1e > compound 1n > compound 1g (most hydrophilic). All five compounds are potent *in vitro* inhibitors of rhPC6, but only the most lipophilic of the compounds (compound 1o) inhibited PC6-dependent decidualization of HESCs, suggesting that the other four compounds failed to inhibit decidualization because of their inability to get into the cell.

### Compound 1o reduces receptivity of Ishikawa endometrial epithelial cells to JAR spheroids

**Figure 5:**
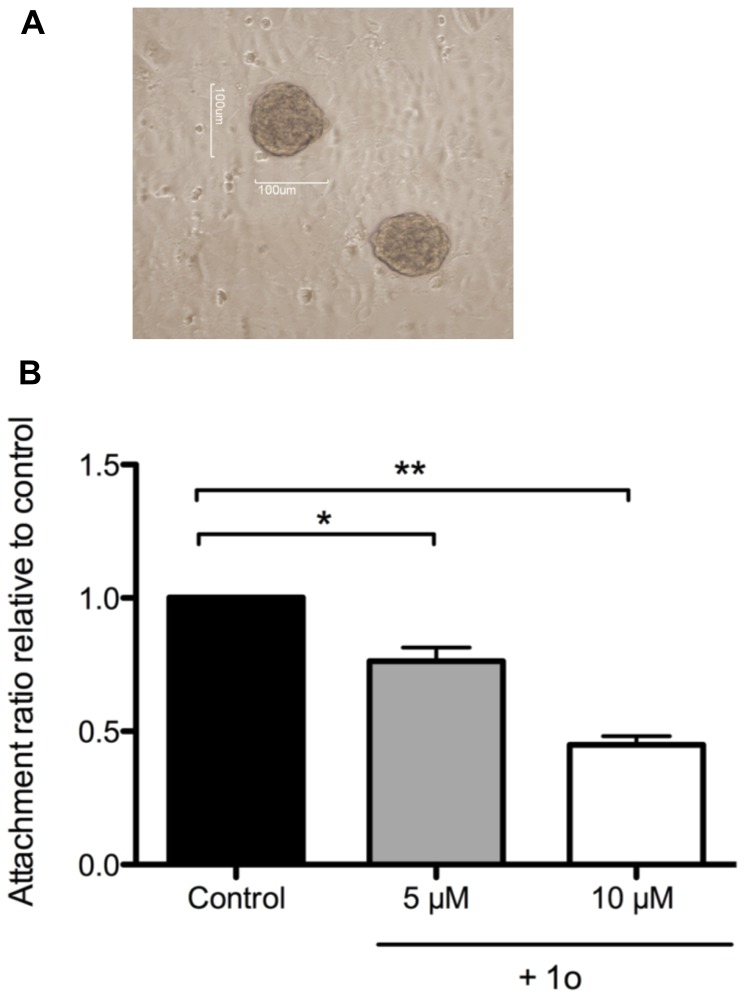
(A) Representative micrograph of JAR spheroids attached to endometrial epithelial cell line Ishikawa monolayer. (B) Inhibition of the receptivity of endometrial epithelial cell line Ishikawa to JAR spheroids by compound 1o in a dose-dependent fashion. The data are expressed as the attachment ratio relative to control. Each bar indicates the mean ± SEM of three independent experiments. *P<0.05; **P<0.01.

We next focused on compound 1o and investigated its effect on the attachment of trophoblast spheroids to endometrial epithelium, employing an *in vitro* implantation model [23]. This is another PC6-dependent cellular process essential for implantation [6], [7]. Human choriocarcinoma JAR cells in suspension were rocked overnight to form spheroids (surrogates for embryos). Endometrial epithelial Ishikawa cell monolayers were formed by culturing them, in the absence or presence of compound 1o for 3 days. JAR spheroids were then co-cultured with Ishikawa monolayers and the number of attached spheroids was calculated (Figure 5A). Compound 1o significantly reduced the receptivity of Ishikawa to JAR spheroids in a dose-dependent manner, with an inhibition of ∼50% at 10 µM and ∼30% at 5 µM (Figure 5B).

### Pro-integrin-αV clevage is inhibited in Ishikawa cells by compound 1o

**Figure 6:**
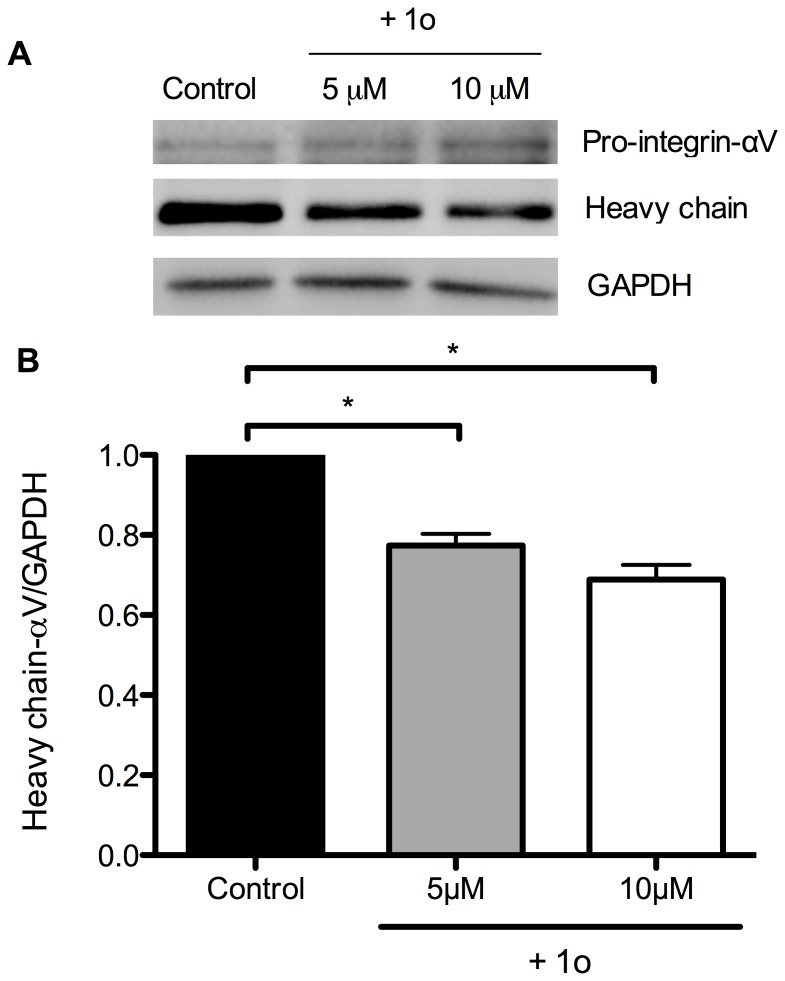
Compound 1o reduces the cleavage of pro-intergrin-αV into its heavy chain. (A) Representative western blot showing the pro-integrin-αV (∼170 kDa) and the heavy chain (130 kDa). (B) Densitometric analysis of the heavy chain of integrin-αV. Densitometric analysis was not performed for pro-integrin-αV as the signals were too weak to be accurately determined. Data were normalized to GAPDH and control and expressed as the mean ± SEM (*n* = 3). *P<0.05.

One of the known mechanisms of PC6 action in regulating endometrial epithelial cell receptivity is through the cleavage of pro-integrin-αV into its functional heavy and light chains [24]. To further establish that compound 1o reduced Ishikawa cell receptivity to spheroid attachment through PC6 inhibition, total cell proteins were analysed for pro-integrin-αV cleavage by western blot. Although both the proform and the heavy chain of integrin-αV were detected in all cell lysates, the relative amount of each form was clearly different between control and compound 1o-treated cells (Figure 6). The heavy chain was reduced whereas the pro-integrin-αV was increased in cells treated with compound 1o compared with controls. This is consistent with PC6 cleavage of the pro-integrin-αV into its functional forms being inhibited by compound 1o.

### Compound 1o is superior to C-30k-PEG Poly R in the inhibition of endometrial epithelial receptivity

**Figure 7:**
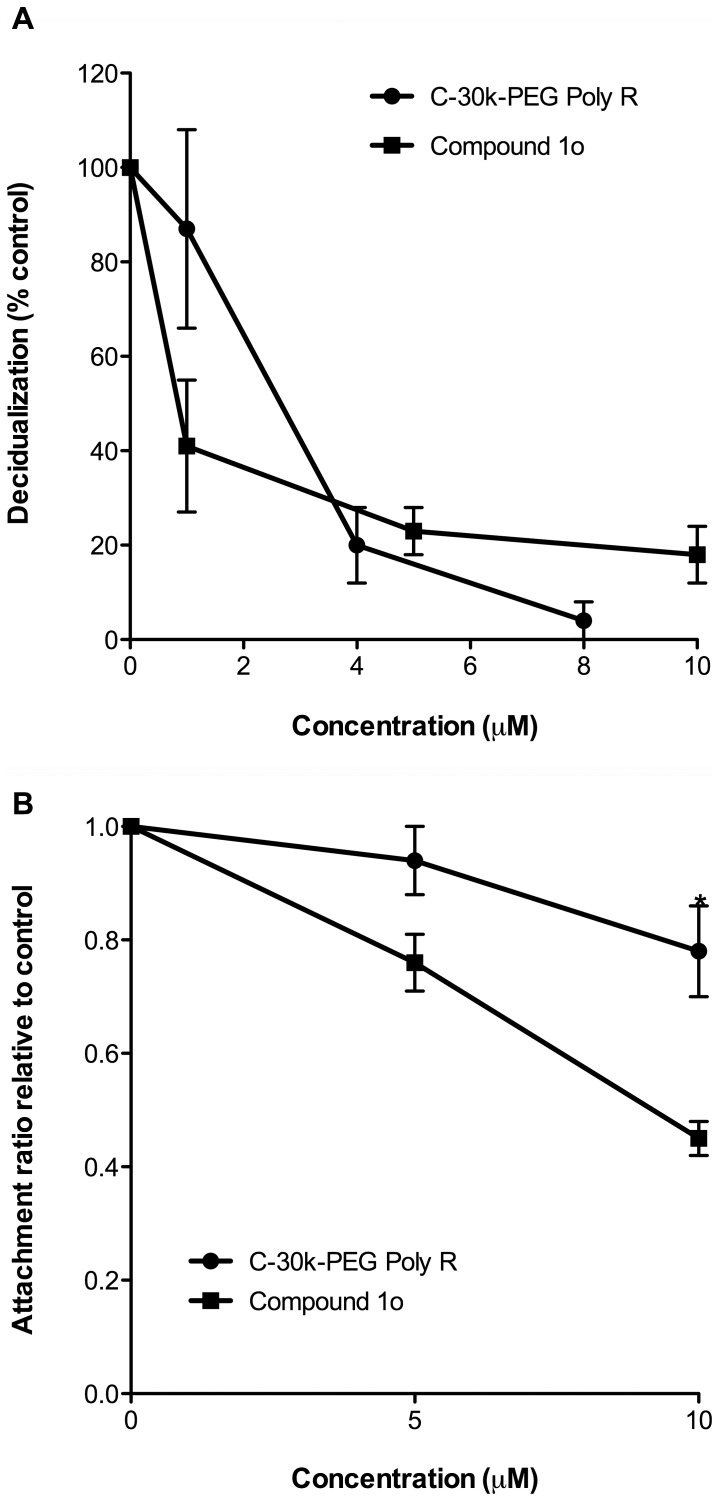
Comparison of compound 1o and C-30k-PEG Poly R in the inhibition of (A) decidualisation of primary HESCs, and (B) Ishikawa cell receptivity to trophoblast spheroid attachment. Data of C-30k-PEG Poly R was previously published in [9]. Each value represents mean ± SEM of three independent experiments. *P<0.05.

This study identified compound 1o as the most potent synthetic small molecule PC6 inhibitor to inhibit PC6-dependent cellular processes essential for embryo implantation. Our previous publication showed C-30k-PEG Poly R as a potent peptide-based PC6 inhibitor [9]. We thus compared these two different types of PC6 inhibitors compound 1o and C-30k-PEG Poly R, for their potency in inhibiting stromal cell decidualization and epithelial receptivity. While both equally inhibited decidualisation of HESCs in a dose-dependent manner (Figure 7A), C-30k-PEG Poly R was significantly less potent than compound 1o in inhibiting Ishikawa cell receptivity to JAR spheroids (Figure 7B). Compound 1o inhibited spheroid attachment (or epithelial receptivity) in a clear dose-dependent manner (0.76 at 5 µM vs 0.45 at 10 µM, p<0.01), and was significantly more inhibitory than C-30k-PEG Poly R (Figure 7B).

## Discussion

PC6 plays a crucial role in embryo implantation and HIV infection; it is therefore highly desirable to develop inhibitors of PC6 for potential non-hormonal female contraceptives that could also protect women from HIV. In the ongoing search for PC6 inhibitors with appropriate physiochemical characteristics for therapeutic applications, we investigated five synthetic small molecule compounds that had been previously reported as inhibitors of furin, another PC member [15]. Our studies revealed that all five compounds (1e, 1f, 1g, 1n and 1o) were potent inhibitors against rhPC6 *in vitro* and they were able to adopt similar binding modes in the hPC6 active site. However, the functional studies by *in vitro* cell-based model demonstrated that only compound 1o was able to inhibit decidualization of HESCs. Prediction of lipophilicity, a physiochemical property related to a compound's ability to cross cellular membranes, revealed that compound 1o was distinct in lipophilicity, being the most lipophilic. Compound 1o was further demonstrated to be potent in inhibiting the receptivity of human endometrial epithelial cells for trophoblast spheroid attachment in an *in vitro* human cell-based model.

It is well established that PC6 is the only PC member that is up-regulated during decidualization, and knockdown of PC6 production by morpholino antisense oligonucleotides in mice *in vivo* resulted in inhibition of decidualization and pregnancy failure [4], [11]. Although compound 1o can inhibit furin and possibly other PC members [15], the inhibitory effect of the compound on decidualization of HESCs was PC6 specific as only PC6 is involved in decidualizaiton [8]. The lack of activity displayed by the other four compounds is likely to be attributed to their poor lipophilicity. Lipophilicity is a key factor that determines how well a molecule can pass through cell membranes [26]. The data presented here suggests that compound 1o has the ideal lipophilicty to cross the cell membrane and reach its site(s) of action, although the exact cell localization of the compound is yet to be determined.

The drug efficiency of compound 1o in the inhibition of PC6 was further evidenced by its ability to significantly reduce the receptivity of endometrial epithelial cells. It is established that PC6 is up-regulated in the human endometrium specifically at the time of epithelial receptivity [6]. The critical role of PC6 in receptivity has been demonstrated by a significant reduction in the attachment of mouse blastocysts to endometrial epithelial cells after specific knockdown of PC6 by small interfering RNA [6]. Furthermore, PC6 regulation of receptivity has been validated in the human endometrium *in vivo* in fertile and infertile women [6]. Endometrial PC6 plays a central role in the post-translational cleavage of pro-integrins (including αV) for blastocyst attachment and adhesion at the commencement of implantation [24]. Compound 1o inhibition of PC6 reduced the cleavage of pro-integrin-αV into its subunits, suggesting that one of the mechanisms of compound 1o inhibition of receptivity is through inhibiting PC6 cleavage of pro-integrins.

In conclusion, our studies have discovered that compound 1o is a potent PC6 inhibitor with potential pharmaceutical properties to inhibit embryo implantation. In addition, compound 1o showed superior potency than C-30k-PEG Poly R (representing a potent peptide PC6 inhibitor) in the inhibition of spheroid attachment in Ishikawa cell. This suggests that PC6 inhibitors in the format of small molecules could have advantages over peptide inhibitors. In both pharmaceutical and academic research, there have been increasing emphases and demand on cell-based assays to reduce the costly failure of drug development in late stages. Here, we highlight the importance of human cell-based functional assays to investigate drug efficiency. These assays provide invaluable information and demonstrate that physicochemical properties of drugs such as lipophilicity must be investigated in addition to biochemical assays; otherwise highly potent drugs selected based on biochemical characteristics may not be necessarily useful. While further studies in animal models are yet to be performed, our data showed for the first time the potential of a non-peptide small molecule PC inhibitor for the development of contraceptives.
